# Suspensory Materials for Surgery of Blepharoptosis: A Systematic Review of Observational Studies

**DOI:** 10.1371/journal.pone.0160827

**Published:** 2016-09-15

**Authors:** Elena Pacella, Daniele Mipatrini, Fernanda Pacella, Giulia Amorelli, Andrea Bottone, Gianpaolo Smaldone, Paolo Turchetti, Giuseppe La Torre

**Affiliations:** 1 Department of Sense Organs, Faculty of Medicine and Dentistry, Sapienza University of Rome, Rome, Italy; 2 Department of Public Health and Infectious Diseases, Faculty of Pharmacy and Medicine, Sapienza, University of Rome, Rome, Italy; 3 National Institute for Health, Migration and Poverty (INMP/NIHMP), Rome, Italy; Universita degli Studi di Firenze, ITALY

## Abstract

**Background:**

Frontalis suspension surgery is considered the procedure of choice in cases of blepharoptosis. Among all the materials used in this type of surgery, ophthalmic and plastic surgeons prefer to use autologous Fascia Lata. However, during years, other autogenous and exogenous materials have been introduced.

**Objectives:**

The aim of this study was therefore that of systematically reviewing the functional results and the rate of complications of different synthetic materials, as compared to autogenous Fascia Lata. The primary objective was to determine the rates of Successful Surgeries (SSs) of these materials. The secondary objective was to assess the onset of complications. The following materials were investigated: Fascia Lata, Mersilene, polytetrafluoroethylene (PTFE) and Silicon.

**Data Source and Methods:**

Following the Prisma procedure, on January 30^th^, 2016 we used the following electronic databases to select the studies: MEDLINE and Scopus.

**Results:**

The search strategy retrieved 48 publications that met the eligibility criteria of the systematic review. All studies were non-comparative. PTFE (n = 5) showed the best rate of SSs among the materials compared (statistically significant). Surgeries performed with autogenous Fascia Lata (n = 19) had a 87% rate of success those performed with Mersilene (n = 12)had 92% and those performed with Silicon (n = 17)88%. PTFE had the best outcome, with 99% success rate. As for complications, surgeries performed with PTFE had a higher rate of suture infections (1.9%) as compared to Fascia Lata, but lower incidence for all other complications.

**Conclusions:**

Although most studies were good quality cohort studies, the overall quality of this evidence should be regarded as low due to their non-comparative design. Our data suggest that PTFE seems to be the most valid alternative material for frontalis suspension surgery, with low recurrence rates and good cosmetic and functional results.

## Introduction

Blepharoptosis is an abnormal low-lying upper eyelid margin with the eye in primary gaze and represents one of the most challenging problem for plastic and oculoplastic surgeons.

Blepharoptosis can ensue from several congenital or acquired conditions [1] and may cause, depending on the age at onset, an almost complete obscuration of the visual axis. In addition, in younger age groups, blepharoptosis is also causing aesthetic concerns, with problems of permanent deprivation, amblyopia and bizarre head posture [2]. It is therefore fundamental to perform a surgery ensuring the best outcome and long–lasting results to the subject affected.

At present, the best surgical practice in cases of ptosis associated with poor levator function (i.e. levator function <4 mm) is the frontalis suspension surgery. The aim of the procedure is to create a connection between the frontalis muscle and the tarsal plate, in order to allow the lid to mobilize in an upward movement when the muscle contracts. Originally described by Payrin in 1909 [3] and Wright in 1922[4], in 1956 this surgery was further developed by Crawford who introduced the use of autogenous Fascia Lata as the standard sling material in children older than three years of age [5].

Nowadays, although autologous Fascia Lata is still the preferred material used for the aforementioned surgery, several other autogenous or exogenous materials have been proposed for the restoration of the deficit of the levator palpebrae [6].

Thus, in this study we aimed at systematically review the functional results and the rate of complications of three different synthetic materials introduced in the surgical practice over years: mersilene mesh, silicon rods and polytetrafluoroethylene (PTFE). Specifically, we compared the results obtained in the surgical practice with these materials to those of autogenous Fascia Lata, which is still the most preferred and adopted material by ophthalmic and plastic surgeons in the management of ptosis related to poor muscle function.

## Methods

### Search strategy, study selection and data extraction

On January 30^th^ 2016, pertinent articles were identified by using the following databases: MEDLINE and Scopus.

We adopted the following search strategies: (Blepharoptosis) AND (frontalis sling OR frontalis suspension OR surgery) AND (mersilene OR polytetrafluoroethylene (PTFE) OR Fascia Lata OR silicon) from January 1989 to 2016. Other articles were identified by analyzing the citations reported in the studies found. Papers resulting from the two databases were matched and duplicates were excluded.

A first screening process for title and abstract was performed by two independent researchers.

After that, we adopted the following inclusion criteria: (a) studies where more than 9 surgeries were performed and monitored; (b) English written; (c) at least 30 days of follow-up; and (d) studies reporting at least one outcome rate (recurrence and/or complications).

Subsequently, the full texts of selected studies were evaluated to confirm the adherence to eligibility criteria. Only cohort studies were considered eligible for inclusion. Randomized Controlled Trials (RCTs), case studies and other study designs were excluded.

Two independent researchers evaluated the papers and extracted the data. Controversies on data extraction were solved by discussion between them, and, in case of disagreement, by the judgment of a third researcher.

### Information selected and definition of outcome measures

Information on the year of publication, the country where the studies took place, the sample size and characteristics (age and gender) was collected.

Information on the cause of blepharoptosis, the material used, the effectiveness of the surgeries performed, the rates of specific pathologic conditions arisen after surgery was also collected.

The first outcome of our search was the rate of Successful Surgeries (SSs), the second outcome was the Overall Rate of Complications (ORC), and the third the rate of each single complication arisen after surgery. We defined as “SSs” the surgeries not requiring a re-intervention regardless of the onset of complications, and not leading to relapse or hesitating in hypo-correction. ORC were defined as the sum of all complications reported in each single study.

### Risk of Bias in individual studies

The risk of bias was assessed by the evaluation of the quality of the included studies. Two independent researchers assessed the Newcastle-Ottawa score for cohort studies [7].

### Summary measures

The pooled SSs prevalence for each material was calculated. The pooled prevalence of ORC and of each single pathologic condition caused by the surgery were also calculated.

### Heterogeneity, risk of bias, meta-regression and data reporting

Statistical heterogeneity was assessed by the Q Cochrane test and I^2^ test

The model adopted for the statistical analysis was a random-effect model.

The pooled prevalence was reported with 95% Confidence Interval (95%CI). Publication bias was measured by the Begg funnel plot [8]. All the Forest plots and the Begg Funnel plots are shown in Supplemental electronic files.

Meta-regression analysis was performed in order to assess whether year of publication, mean age of the sample, the gender distribution, the length of follow-up or the quality of studies could affect the rate of SSs and ORC. Statistical analyses were performed with Stat Direct Software (pooled incidence) and SPSS (meta-regression) Software. Data were reported according to the PRISMA protocol [9].

## Results

### Study selection

**Fig 1:**
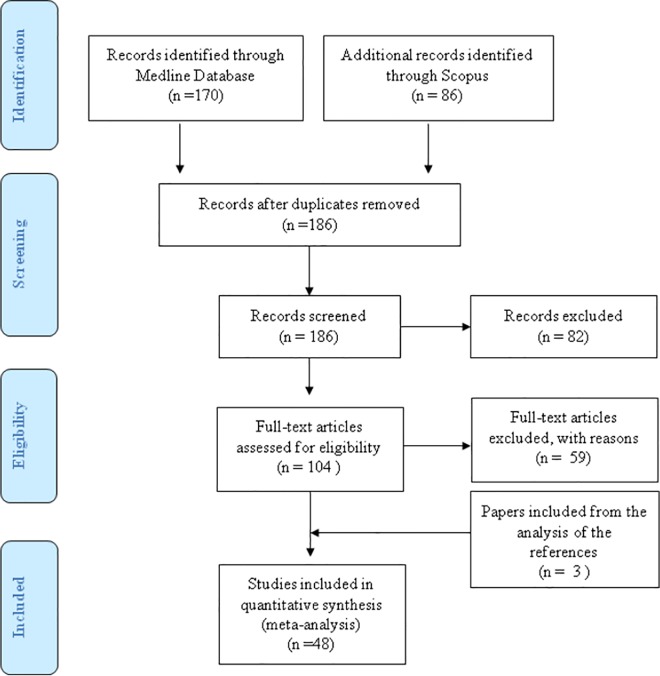
Flow chart of study selection.

The search strategy retrieved 256 records in two different databases. After deduplication and examination of the titles and abstracts, 152 studies were excluded from the review process (S1 Table). 105 full-text copies of the remaining studies were obtained and subjected to further evaluation. 59 of these studies were excluded and the reasons for exclusion were annotated. At the end of the process, 48 publications meeting the eligibility criteria were selected for review (Fig 1). The number of studies included for Fascia Lata were 19 [10–28], for Mersilene 12 [29,30,22,31,32,25,33–38], for PTFE 5 [39–41,26,42] and for Silicon 17 [43–52,13,53,54,20,55–57].

### Characteristics of the included studies and quality assessment

Table 1 shows characteristics of the studies included [15,29,30,32,34–36,38–41,51]. The time range of the selected studies was 1990[38]-2015[35]. Results of the quality assessment performed through the Ottawa-Newcastle scale are shown in Table 1.

**Table 1:** Included studies.

First author (year)	Design	N. of patients	N. of operations	Material	Quality
Shimizu (2015) [16]	Cohort/Case series	11	11	Fascia Lata	7
Woo (2014) [17]	Cohort/Case series	82	101	Fascia Lata	6
Suh (2013) [15]	Cohort/Case series	47	60	Fascia Lata	7
Arajy (2012) [12]	Cohort/Case series	51	69	Fascia Lata	7
Evereklioglu (2012)[18]	Cohort/Case series	18	26	Fascia Lata	6
Sokol (2011) [13]	Cohort/Case series	-	156	Fascia Lata	6
Bilgin (2010) [14]	Cohort/Case series	35	43	Fascia Lata	7
Philandrianos (2010) [19]	Cohort/Case series	9	12	Fascia Lata	6
Lee (2009) [20]	Cohort/Case series	63	91	Fascia Lata	6
Yoon (2009) [11]	Cohort/Case series	239	324	Fascia Lata	8
Cates (2008) [21]	Cohort/Case series	13	21	Fascia Lata	6
Salour (2008) [22]	Cohort/Case series	10	15	Fascia Lata	6
Bagheri (2007) [23]	Cohort/Case series	19	30	Fascia Lata	6
DeMartelaere (2007) [24]	Cohort/Case series	25	48	Fascia Lata	6
Leibovitch(2003) [10]	Cohort/Case series	9	14	Fascia Lata	6
El-Toukhy (2001) [25]	Cohort/Case series	24	34	Fascia Lata	6
Wesserman (2001) [26]	Cohort/Case series	43	102	Fascia Lata	6
Khwarg (1999) [27]	Cohort/Case series	24	27	Fascia Lata	6
Deenstra (1996) [28]	Cohort/Case series	81	81	Fascia Lata	6
Chong (2010) [29]	Cohort/Case series	10	10	Mersilene	8
Hafez (2008) [30]	Cohort/Case series	30	50	Mersilene	6
Salour (2008) [22]	Cohort/Case series	10	16	Mersilene	6
Mehta (2005) [31]	Cohort/Case series	20	32	Mersilene	6
Sharma (2003) [32]	Cohort/Case series	41	71	Mersilene	7
El-Toukhy (2001) [25]	Cohort/Case series	32	46	Mersilene	6
Kemp (2001) [33]	Cohort/Case series	20	29	Mersilene	6
Lam (1997) [34]	Cohort/Case series	10	10	Mersilene	5
Can (1996) [35]	Cohort/Case series	22	23	Mersilene	6
Gabrieli (1996) [36]	Cohort/Case series	14	20	Mersilene	6
Hintschich (1995) [37]	Cohort/Case series	54	76	Mersilene	6
Downes (1990) [38]	Cohort/Case series	15	23	Mersilene	5
Hayashi (2013) [39]	Cohort/Case series	31	42	PTFE	8
Nakauchi (2013) [40]	Cohort/Case series	20	27	PTFE	8
Wei (2009) [41]	Cohort/Case series	96	130	PTFE	8
Wesserman (2001) [26]	Cohort/Case series	43	102	PTFE	6
Steinkogler (1993) [42]	Cohort/Case series	26	37	PTFE	6
Bansal (2015) [43]	Cohort/Case series	25	38	Silicon	6
Kim (2015) [44]	Cohort/Case series	-	98	Silicon	6
Nucci (2015) [45]	Cohort/Case series	22	44	Silicon	6
Razavi (2014) [46]	Cohort/Case series	44	70	Silicon	6
Rizvi (2014) [47]	Cohort/Case series	46	56	Silicon	6
Buttanri (2013) [48]	Cohort/Case series	56	80	Silicon	6
Allen (2012) [49]	Cohort/Case series	31	62	Silicon	7
Friedhofer (2012) [50]	Cohort/Case series	112	141	Silicon	6
Van Sorge (2012) [51]	Cohort/Case series	69	101	Silicon	7
Ali (2011) [52]	Cohort/Case series	33	35	Silicon	6
Sokol (2011) [13]	Cohort/Case series	-	11	Silicon	6
Lamont (2010) [53]	Cohort/Case series	26	42	Silicon	6
Bernardini (2009) [54]	Cohort/Case series	10	16	Silicon	6
Lee (2009) [20]	Cohort/Case series	60	90	Silicon	6
Lelli (2009) [55]	Cohort/Case series	33	51	Silicon	7
Fogagnolo (2008) [56]	Cohort/Case series	22	22	Silicon	6
Carter (1996) [57]	Cohort/Case series	35	61	Silicon	6

This scale was adopted to evaluate the potential bias within the studies. All the studies scored 5 points or above. Quality score was included as covariate into the meta-regression to assess whether quality affected the results of the studies.

The prevalence of SSs with Fascia Lata was 87% with 95%CI (range: 80–93%). The SSs prevalence of Mersilene was higher: 92% with 95% CI (range 87–96%). The highest prevalence of SSs was observed with the procedures utilizing PTFE: 99% with 95%CI (range 98–100%) while the prevalence of SSs found in surgeries performed with silicon was 88%with 95% CI (83–91%).

The lower limit of 95% CI of SSs performed with PTFE was above all the upper limits of 95% CI of SSs performed with other materials, thus the PTFE has statistically significant higher success rate than other materials.

### Pooled cumulative incidence of Complications (Cs)

The cumulative incidence of Cs with Fascia Lata was 25% with 95%CI ranging between 14% and 39%. For Mersilene the Cs cumulative incidence was 17% with 95%CI (range 9–27%). Again, PTFE showed the best results, as the CS prevalence was only 5% (range 0.6–13%). Silicon showed a Cs rate of 15% with 95% CI (range 9–23%). PTFE has significant lower incidence of Cs in comparison with Fascia lata but not with Mersilene and silicon.

### Prevalence of single complications

Table 2 shows the single complications for each material. In surgeries performed utilizing Fascia Lata, exposure keratitis was reported in 3.8% of cases (95%CI = 1.5–7.3), Lagophtalmos in 2.5% cases, asymmetry and amblyopia in 2.1% (95%CI = 0-7- 4.1), Entropion in 2% of cases, overcorrection in 1.2% (95%CI = 0.4–2.7), and strabismus in 1.2% (95%CI = 0.3–2.8). Suture infections occurred in 1% (95%CI = 0.5–1.6). All the remaining complications occurred in less than 1% of surgeries.

**Table 2:** Pooled percent prevalence of Successful Surgeries (SSs) and cumulative incidence of complications for each material considered. CI = confidence interval.

	Fascia Lata	Mersilene	PTFE	Silicon
	% (95%CI)	% (95%CI)	% (95%CI)	% (95%CI)
**Succesfull Surgeries (SSs)**	87 (80–93)	92 (87–96)	99 (98–100)	88 (83–91)
**Tot complications (Cs)**	25 (14–39)	17 (9–27)	5 (0.6–13)	15 (9–23)
**Suture infection**	1 (0.5–1.6)	1.4 (0.4–3.0)	1.9 (0.2–5.4)	0.6 (0.2–1.2)
**Lagophthalmos**	2.5 (0.7–5.3)	3.6 (0.8–8.2)	0.3 (0.0–1.2)	2.2 (0.6–4.8)
**Asymmetry**	2.1 (0.7–4.1)	2.4 (0.7–5.1)	1.5 (0.0–5.3)	1.6 (0.4–3.5)
**Granuloma**	0.5 (0.2–0.9)	2.3 (0.8–4.5)	1.2 (0.0–3.7)	1.0 (0.4–1.8)
**Herniation**	0.4 (0.1–0.8)	1.5 (0.5–3.1)	0.5 (0.0–1.5)	2.0 (1.0–3.4)
**Overcorrection**	1.2 (0.4–2.7)	1.0 (0.3–2.2)	0.3 (0.0–1.2)	0,7 (0.3–1.3)
**Hypertrophic scar**	0.8 (0.3–1.6)	-	-	-
**Preseptal cellulitis**	0.3 (0.0–0.7)	0.8 (0.0–1.8)	0.3 (0.0–1.2)	0.6 (0.2–1.2)
**Entropion**	2.0 (0.8–3.6)	0.7 (0.1–1.7)	0.3 (0.0–1.2)	0.5 (0.2–1.0)
**Abscess**	0.4 (0.1–0.8)	0.8 (0.2–1.9)	0.3 (0.0–1.2)	0.5 (0.2–1.1)
**Amblyopia**	2.1 (0.0–7.3)	0.7 (0.1–1.7)	0.3 (0.0–1.2)	0.4 (0.0–0.8)
**Strabismus**	1.2 (0.3–2.8)	0.7 (0.1–1.7)	0.3 (0.0–1.2)	0.4 (0.0–0.8)
**Exposure keratitis**	3.8 (1.5–7.3)	3.2 (0.9–6.6)	0.3 (0.0–1.2)	3.0 (0.6–7.2)

When compared to the Fascia Lata group, surgeries performed with Mersilene showed higher percentages of Lagophthalmos (3,6%; 95%CI: 0,7–5,3), asymmetry (2,4%; 95%CI: 0,4–5.1), granuloma (2.3%; 95%CI = 0.8–4.5), herniation (1.5%; 95%CI: 0.5–3.1) and suture infection (1.4%; 95%CI: 0.4–3.0). On the contrary, lower percentages of overcorrection (1%; 95%CI = 0.4–2.7), entropion (0.7%; 95%CI = 0.1–1.7), strabismus (0.7%; 95%CI = 0.1–1.7) and exposure keratitis (3.2%; 95%CI = 0.9–6.6) were observed.

Interventions performed with PTFE showed higher percentages of the suture infections (1.9%; 95%CI = 0.2–5.3) as compared to Fascia Lata, but lower incidence of all other complications.

When compared to Fascia Lata, interventions utilizing Silicon had higher rates of herniation (2.0%; 95%CI = 1–3.4) but lower incidence of all other complications.

### Meta regression

A meta-regression analysis for the two main outcomes (SSs and overall rate of complications) was performed on all studies included and separately on studies evaluating Fascia Lata and Silicone (S1 Fig). The number of studies evaluating PTFE (5) [26,39–42] and Mersilene (12) [29,30,22,31,32,25,33–38] did not allow to perform the meta-regression in these groups. The year of publication, the mean age of the sample, the proportion of male among patients, the mean follow-up and the quality of the studies were used as covariates in the analysis.

The results of meta-regression of all studies included (Table 3) showed that year of publication, the proportion of male in the sample and the quality of the studies negatively correlate with the prevalence of SSs while the mean age of the sample does not. The proportion of male positively correlates with the rate of complications while the mean follow-up and the quality of the study negatively correlates with overall rate of complications (ORC).

**Table 3:** Meta-regression for the outcomes Succesfull Surgeries (SSs) and Overal Rate of Complications.

**Successful operations**	**All materials**		**Fascia lata**		**Silicon**	
	β	P	β	P	β	P
Year of publication	-0.10	<0.1	1.06	<0.01	-0.60	<0.01
Mean age	-0.04	0.17	0.48	<0.01	-0.67	<0.01
Male proportion	-0.35	<0.01	-0.91	<0.01	-0.66	<0.01
Mean follow-up	0.00	0.85	0.09	0.01	0.14	0.01
Study quality	-0.29	<0.01	-0.18	<0.01	-0.06	0.46
R2	0.24		0.63		0.35	
**Complications**	**All materials**		**Fascia lata**		**Silicon**	
	β	P	β	P	β	P
Year of publication	0.03	0.33	0.71	<0.01	-0.32	<0.01
Mean age	0.04	0.12	0.37	<0.01	0.09	0.24
Male proportion	0.25	<0.01	-0.17	<0.01	0.27	<0.01
Mean follow-up	-0.10	<0.01	0.14	<0.01	-0.42	<0.01
Study quality	-0.09	<0.01	-0.32	<0.01	0.15	0.03
		0.06		0.35		0.41

In studies evaluating Fascia, Lata (19) [10–28] the year of publication, the mean age of the sample and the mean follow-up positively correlate with the SSs and the ORC, while the proportion of males and the study quality negatively correlates with both outcomes. In studies evaluating Silicon (17) [43–52,13, 53,54,20,55–57], the year of publication, the mean age and the proportion of males negatively correlate with the SSs while the mean follow-up positively correlates the SSs. The year of publication and the mean follow-up negatively correlates with the ORC while male proportion and the quality of the studies positively correlates with ORC.

## Discussion

The correction of a blepharoptosis is crucial to avoid long term visual consequences during developmental age, such as abnormal head postures and amblyopia. In addition, the level of satisfaction of the patient, i.e. functional and aesthetic outcomes, represents an additional challenge to the surgeon performing ptosis correction. The optimal surgical intervention should not necessitate reoperations, should not lead to the onset of complications and would necessitate the least number of follow-up visits.

In this review, we aimed at evaluating the influence that the choice of the suspension material, either autologous (Fascia Lata) or allogenic (mersilene, PTFE, and silicon) may have on functional results (Successful Surgeries, SSs) and rate of complications. Our analysis showed that the rate of successful surgeries and complications did not significantly vary among these materials, although slight differences were observed. In fact, while patients undergoing ptosis repair with autogenous Fascia Lata had a 90% rate of SSs in the long term (i.e. not requiring new interventions or recurring during the follow-up period), Mersilene and PTFE seem to lead to better outcomes, with a rate of 95 and 99% respectively. Silicon has the lowest potential as a long lasting suspensory material, with a rate of success of 79%.

The advantages and the complications of each materials, as reported in the literature analyzed, are described below.

### Fascia Lata

The advantage of using Fascia Lata consists in the possibility to harvest autologous material [26], thus minimizing the risk of rejection or extrusion of the sling as compared to allogenic sources [57]. For these reasons, Fascia Lata has been considered the optimal material by the majority of surgeons, especially for lid suspension procedures in children under 3 years of age [5]. Recently, Leibovitch addressed the possibility of using Fascia Lata in newborns with congenital ptosis, demonstrating and confirming the feasibility of this procedure even in younger age groups [10]. In addition, Evereklioglu experimented a new minimally invasive ‘kite-tail’ Fascia Lata strip technique, consisting of 3 to 5 cm length of Fascia Lata graft. The first and foremost consequence is the lower rate of complications at the surgical site compared to the traditional technique, with the avoidance of muscle prolapse, haematoma formation and functional disabilities in the early postoperative days [18]. Naugle and coworkers previously reported a favorable outcome using Fascia Lata in two 3-year-old patients and in a 2-year-old child affected by congenital ptosis [60]. However, despite its highest biocompatibility and the lowest rate of sling extrusion [30,34,35], Fascia Lata is difficult to harvest in infants, and the possibility of permanent thigh scars makes it an unattractive choice to the parents [10,33,63].

### Mersilene

To obviate the lack of suitable alternatives in younger patients with blepharoptosis, novel eterologous materials were introduced in oculoplastic surgery by the end of the eighties. The research was focused on finding synthetic sling materials with the same biocompatibility and durability of Fascia Lata, and eliminating the need to perform a double procedure at two different surgical sites. In 1989, Downes and Collins introduced for the first time the use of Mersilene mesh in blepharoptosis surgery, with success rates similar to autogenous materials [64]. Since then, Mersilene has been widely accepted as a valid alternative, being suitable even for children under 3 years of age.

Chong [29] reported only a 10% failure rate with Mersilene mesh in infants requiring early correction of blepharoptosis, posing new promising possibilities for younger patients and overcoming the issue of the diffuse incapability of the ophthalmic surgeon to harvest autologous material from a site far from his usual area of competence[25]. Moreover, the use of Mersilene is encouraged by other advantages, such as shorter surgical times and reduced number of multiple specialists within the surgical team.

Nonetheless, other studies revealed that the major complications associated with the use of mersilene are the relatively high incidence of sling extrusion, granuloma formation [65] and infection [36]. Only minor corneal complaints were reported that just required the use of topical lubricants.

In such cases, the use of a reversible allogenic material and a minor degree of overcorrection is advisable, especially in older patients with tearing problems or poor or absent Bell’s phenomenon.

Our analysis revealed that the use of Mersilene Mesh, compared to other materials, leads to higher rates of lagophthalmos (3.1%), asymmetry (2.8%) and granuloma (2.1%) but the lower rates of suture infection (1.0%).

### Polytetrafluoroethylene (PTFE)

Among the materials investigated in the present review, our analysis revealed that PTFE shows the best results, as for percentage of successful surgeries (99%;) and the overall complication rate (5%). Concerning the SSs PTFE is significantly better than other materials.

The major concern related to the use of PTFE is the rate of infectious complications or granuloma formation. For this complication PTFE shows the highest rate in comparison with the other materials (1.9%). Nonetheless, according to our results, PTFE shows the lowest rates for all the other complications examined: asymmetry, granuloma, overcorrection, hypertrophic scar, preseptal cellulitis, entropion, abscess, amblyopia, strabismus, and exposure keratitis.

### Silicon

In our examination, silicon was the least successful of all synthetic materials, with a 88% rate of successful surgeries (SSs). Silicon was first used by Tillett and Tillett in 1966 [42,66] as a pioneer sling material for blepharoptosis with poor levator function. Since then, silicon has been widely used in several surgical series, reporting a recurrence rate ranging from 7% to 44% [40,54–68].

This wide range of postoperative recurrence rates among the studies we included in the analysis especially for silicone might be related to the effect of different surgical designs, postoperative follow-up periods and blepharoptosis etiologies. Particularly, compared to other materials, silicon seems to be useful in the management of patients affected by myogenic ptosis and poor Bell’s phenomenon (myasthenia gravis, third nerve palsy, CPEO [Chronic Progressive External Ophthalmoplegia])[20]. In fact, silicon, given its wide availability and elastic properties, results more appropriate in patients with high level of corneal exposure that requires frequent adjustments of the sling height [6,69]. In contrast, other non-stretchable materials, like Fascia Lata and polytetrafluoroethylene, are preferred in young children in whom a long term solution is advisable and aesthetic outcome is essential.

As for complications lagophtalmos and herniation were the most frequent. In the literature, it is also reported a case of bilateral granuloma formation, probably caused by Candida or Atypical Micobacteria, in a 5-year-old child requiring bilateral blepharoptosis surgery and multiple revisions at the surgical site [70].

### Limitations

The study present some limitations. The first is the lack of a direct comparison among the different materials and of a measure of association for the comparison of the outcomes. The majority of the studies take into account only one materials and do not compare outcomes among two or more materials. Performing a network meta-analysis to provide association measure would be misleading because we would compare completely different populations and surgical equips.

The second limitation is the lack of information on the surgical technique. Different results might be due to different techniques adopted.

## Conclusion

Although most studies were good quality cohort studies, the overall quality of this evidence should be regarded as low due to their non-comparative design. PTFE shows the highest rate of SSs, statistically significant, and the lower rate of complications. The results of this study suggest that PTFE is the material with lowest recurrence rates and good cosmetic and functional results for frontalis suspension surgery.

## Supporting Information

S1 PRISMA Checklist PRISMA 2009 Checklist. (DOCX)

S1 Fig Forest Plots. (DOCX)

S1 Table Paper excluded. (DOCX)
