# Supplementary material for: Suspensory Materials for Surgery of Blepharoptosis: A Systematic Review of Observational Studies
Source: PLoS One. 2016 Sep 15;11(9):e0160827. doi: 10.1371/journal.pone.0160827 (PMC5025102; doi:10.1371/journal.pone.0160827)
Supplement: S1 Fig — (DOCX) [file pone.0160827.s002.docx]

**SUCCESSFUL SURGERIES (SSs)**

Fascia lata

Non-combinability of studies

Cochran Q = 211,39075 (df = 18) P < 0,0001

Moment-based estimate of between studies variance = 0,169585

I² (inconsistency) = 91,5% (95% CI = 88,6% to 93,3%)

Random effects (DerSimonian-Laird)

Pooled proportion = 0,874116 (95% CI = 0,800684 to 0,932607)

Bias indicators

Begg-Mazumdar: Kendall's tau = -0,321637 P = 0,049

Egger: bias = -3,234202 (95% CI = -5,715478 to -0,752926) P = 0,0137

Harbord: bias = 1,657459 (92,5% CI = -1,454877 to 4,769795) P = 0,3266

Mersilene

Non-combinability of studies

Cochran Q = 29,674753 (df = 11) P = 0,0018

Moment-based estimate of between studies variance = 0,050463

I² (inconsistency) = 62,9% (95% CI = 16,7% to 78,6%)

Random effects (DerSimonian-Laird)

Pooled proportion = 0,922087 (95% CI = 0,87192 to 0,960619)

Bias indicators

Begg-Mazumdar: Kendall's tau b = -0,507692 P = 0,0275

Egger: bias = -1,763741 (95% CI = -4,045798 to 0,518316) P = 0,1158

Harbord: bias = 1,656235 (92,5% CI = -1,556661 to 4,86913) P = 0,3298

PTFE

Non-combinability of studies

Cochran Q = 5,068406 (df = 4) P = 0,2804

Moment-based estimate of between studies variance = 0,004271

I² (inconsistency) = 21,1% (95% CI = 0% to 71,1%)

Random effects (DerSimonian-Laird)

Pooled proportion = 0,991707 (95% CI = 0,976705 to 0,99915)

Bias indicators

Begg-Mazumdar: Kendall's tau = -0,8 P = 0,0167 (low power)

Egger: bias = -0,645615 (95% CI = -1,529317 to 0,238087) P = 0,1026

Harbord: bias = -2,340823 (92,5% CI = -6,467356 to 1,78571) P = 0,2257

Silicone

Non-combinability of studies

Cochran Q = 62,227658 (df = 16) P < 0,0001

Moment-based estimate of between studies variance = 0,048351

I² (inconsistency) = 74,3% (95% CI = 55,8% to 82,9%)

Random effects (DerSimonian-Laird)

Pooled proportion = 0,876105 (95% CI = 0,832047 to 0,914282)

Bias indicators

Begg-Mazumdar: Kendall's tau = -0,308824 P = 0,0762

Egger: bias = -2,904876 (95% CI = -6,160498 to 0,350746) P = 0,0766

Harbord: bias = 2,763969 (92,5% CI = -0,085793 to 5,613732) P = 0,0833

**Total complications**

Fascia lata

Non-combinability of studies

Cochran Q = 457,400362 (df = 18) P < 0,0001

Moment-based estimate of between studies variance = 0,385311

I² (inconsistency) = 96,1% (95% CI = 95,2% to 96,7%)

Random effects (DerSimonian-Laird)

Pooled proportion = 0,254428 (95% CI = 0,140225 to 0,389104)

Bias indicators

Begg-Mazumdar: Kendall's tau = 0,461988 P = 0,0052

Egger: bias = 4,473651 (95% CI = -1,100411 to 10,047713) P = 0,1086

Harbord: bias = 1,990366 (92,5% CI = -3,130892 to 7,111625) P = 0,4711

Mersilene

Non-combinability of studies

Cochran Q = 60,483318 (df = 11) P < 0,0001

Moment-based estimate of between studies variance = 0,133714

I² (inconsistency) = 81,8% (95% CI = 67,9% to 88,1%)

Random effects (DerSimonian-Laird)

Pooled proportion = 0,172905 (95% CI = 0,093894 to 0,269871)

Bias indicators

Begg-Mazumdar: Kendall's tau = 0,545455 P = 0,0138

Egger: bias = 3,388693 (95% CI = 2,135487 to 4,641898) P = 0,0001

Harbord: bias = 3,176468 (92,5% CI = -0,572664 to 6,925599) P = 0,1232

PTFE

Non-combinability of studies

Cochran Q = 28,533776 (df = 4) P < 0,0001

Moment-based estimate of between studies variance = 0,098076

I² (inconsistency) = 86% (95% CI = 64,6% to 92,2%)

Random effects (DerSimonian-Laird)

Pooled proportion = 0,049783 (95% CI = 0,005567 to 0,134393)

Bias indicators

Begg-Mazumdar: Kendall's tau = 0,4 P = 0,4833 (low power)

Egger: bias = 2,087826 (95% CI = -0,682716 to 4,858369) P = 0,096

Harbord: bias = 2,274557 (92,5% CI = -9,761086 to 14,310199) P = 0,6473

Silicone

Non-combinability of studies

Cochran Q = 146,383131 (df = 16) P < 0,0001

Moment-based estimate of between studies variance = 0,136371

I² (inconsistency) = 89,1% (95% CI = 84,5% to 91,8%)

Random effects (DerSimonian-Laird)

Pooled proportion = 0,154874 (95% CI = 0,092889 to 0,229223)

Bias indicators

Begg-Mazumdar: Kendall's tau = 0,397059 P = 0,0273

Egger: bias = 3,973758 (95% CI = 2,470073 to 5,477442) P < 0,0001

Harbord: bias = 2,064169 (92,5% CI = -2,908166 to 7,036504) P = 0,4394

**COMPLICATIONS**

**Cellulitis**

Fascia lata

Non-combinability of studies

Cochran Q = 5,192428 (df = 21) P = 0,9998

Moment-based estimate of between studies variance = 0

I² (inconsistency) = 0% (95% CI = 0% to 40,8%)

Random effects (DerSimonian-Laird)

Pooled proportion = 0,00327 (95% CI = 0,000909 to 0,007087)

Bias indicators

Begg-Mazumdar: Kendall's error P > 0,9999

Egger: bias = 0 (95% CI = 0 to 0) P = *

Harbord: bias = 6,13E-18 (92,5% CI = -1,15E-08 to 1,15E-08) P > 0,9999

Egger: bias = -2,822163 (95% CI = -4,963483 to -0,680843) P = 0,0124

Harbord: bias = 1,4701 (92,5% CI = -1,000852 to 3,941051) P = 0,277

Mersilene

Non-combinability of studies

Cochran Q = 3,647914 (df = 11) P = 0,9791

Moment-based estimate of between studies variance = 0

I² (inconsistency) = 0% (95% CI = 0% to 49,8%)

Random effects (DerSimonian-Laird)

Pooled proportion = 0,008036 (95% CI = 0,001749 to 0,018841)

Bias indicators

Begg-Mazumdar: Kendall's tau b = 0,938462 P < 0,0001

Egger: bias = 0,152348 (95% CI = -0,126381 to 0,431078) P = 0,2512

Harbord: bias = 0,444465 (92,5% CI = -0,866003 to 1,754934) P = 0,5156

PTFE

Non-combinability of studies

Cochran Q = 0,434134 (df = 4) P = 0,9796

Moment-based estimate of between studies variance = 0

I² (inconsistency) = 0% (95% CI = 0% to 64,1%)

Random effects (DerSimonian-Laird)

Pooled proportion = 0,003349 (95% CI = 0,000025 to 0,012231)

Bias indicators

Begg-Mazumdar: Kendall's error P > 0,9999 (low power)

Egger: bias = 0 (95% CI = 0 to 0) P = *

Harbord: bias = -1,49E-16 (92,5% CI = * to *) P < 0,0001

Silicone

Non-combinability of studies

Cochran Q = 12,235315 (df = 16) P = 0,7276

Moment-based estimate of between studies variance = 0

I² (inconsistency) = 0% (95% CI = 0% to 44,5%)

Random effects (DerSimonian-Laird)

Pooled proportion = 0,006334 (95% CI = 0,00242 to 0,01208)

Bias indicators

Begg-Mazumdar: Kendall's tau = 0,941176 P < 0,0001

Egger: bias = 0,258204 (95% CI = -0,279247 to 0,795656) P = 0,3221

Harbord: bias = -1,051279 (92,5% CI = -3,550316 to 1,447757) P = 0,4335

**Exposure keratitis**

Fascia lata

Non-combinability of studies

Cochran Q = 118,365525 (df = 18) P < 0,0001

Moment-based estimate of between studies variance = 0,088011

I² (inconsistency) = 84,8% (95% CI = 77,6% to 88,9%)

Random effects (DerSimonian-Laird)

Pooled proportion = 0,03839 (95% CI = 0,014561 to 0,072932)

Bias indicators

Begg-Mazumdar: Kendall's tau = 0,637427 P < 0,0001

Egger: bias = 1,557961 (95% CI = 0,432282 to 2,683639) P = 0,0095

Harbord: bias = 2,260517 (92,5% CI = -1,797371 to 6,318405) P = 0,3055

Mersilene

Non-combinability of studies

Cochran Q = 27,811926 (df = 11) P = 0,0035

Moment-based estimate of between studies variance = 0,045429

I² (inconsistency) = 60,4% (95% CI = 8,9% to 77,4%)

Random effects (DerSimonian-Laird)

Pooled proportion = 0,031766 (95% CI = 0,009708 to 0,065933)

Bias indicators

Begg-Mazumdar: Kendall's tau b = 0,753846 P = 0,0009

Egger: bias = 1,143599 (95% CI = 0,473092 to 1,814106) P = 0,0035

Harbord: bias = 4,081808 (92,5% CI = 0,463382 to 7,700233) P = 0,0489

PTFE

Non-combinability of studies

Cochran Q = 0,434134 (df = 4) P = 0,9796

Moment-based estimate of between studies variance = 0

I² (inconsistency) = 0% (95% CI = 0% to 64,1%)

Random effects (DerSimonian-Laird)

Pooled proportion = 0,003349 (95% CI = 0,000025 to 0,012231)

Bias indicators

Begg-Mazumdar: Kendall's error P > 0,9999 (low power)

Egger: bias = 0 (95% CI = 0 to 0) P = *

Harbord: bias = -1,49E-16 (92,5% CI = * to *) P < 0,0001

Silicone

Non-combinability of studies

Cochran Q = 152,928813 (df = 16) P < 0,0001

Moment-based estimate of between studies variance = 0,143217

I² (inconsistency) = 89,5% (95% CI = 85,3% to 92,1%)

Random effects (DerSimonian-Laird)

Pooled proportion = 0,030152 (95% CI = 0,006027 to 0,071837)

Bias indicators

Begg-Mazumdar: Kendall's tau = 0,647059 P = 0,0001

Egger: bias = 2,592388 (95% CI = 0,561806 to 4,62297) P = 0,0158

Harbord: bias = -2,791923 (92,5% CI = -11,299588 to 5,715742) P = 0,5395

**Suture infection**

Fascia lata

Non-combinability of studies

Cochran Q = 7,246561 (df = 18) P = 0,9879

Moment-based estimate of between studies variance = 0

I² (inconsistency) = 0% (95% CI = 0% to 42,9%)

Random effects (DerSimonian-Laird)

Pooled proportion = 0,010215 (95% CI = 0,00545 to 0,016444)

Bias indicators

Begg-Mazumdar: Kendall's tau = 0,333333 P = 0,049

Egger: bias = -0,063882 (95% CI = -0,43653 to 0,308765) P = 0,722

Harbord: bias = -0,47364 (92,5% CI = -1,079495 to 0,132216) P = 0,1564

Mersilene

Non-combinability of studies

Cochran Q = 13,713774 (df = 11) P = 0,2492

Moment-based estimate of between studies variance = 0,007333

I² (inconsistency) = 19,8% (95% CI = 0% to 59,2%)

Random effects (DerSimonian-Laird)

Pooled proportion = 0,01397 (95% CI = 0,003986 to 0,029894)

Bias indicators

Begg-Mazumdar: Kendall's tau b = 0,876923 P = 0,0001

Egger: bias = 0,496743 (95% CI = -0,1645 to 1,157986) P = 0,1251

Harbord: bias = 0,771657 (92,5% CI = -2,866423 to 4,409737) P = 0,6823

PTFE

Non-combinability of studies

Cochran Q = 10,911312 (df = 4) P = 0,0276

Moment-based estimate of between studies variance = 0,027628

I² (inconsistency) = 63,3% (95% CI = 0% to 84%)

Random effects (DerSimonian-Laird)

Pooled proportion = 0,019413 (95% CI = 0,002112 to 0,053583)

Bias indicators

Begg-Mazumdar: Kendall's tau = 0,4 P = 0,4833 (low power)

Egger: bias = 1,048558 (95% CI = -1,294275 to 3,391391) P = 0,2496

Harbord: bias = -0,161974 (92,5% CI = -7,96079 to 7,636842) P = 0,9591

Silicone

Non-combinability of studies

Cochran Q = 11,775566 (df = 16) P = 0,7593

Moment-based estimate of between studies variance = 0

I² (inconsistency) = 0% (95% CI = 0% to 44,5%)

Random effects (DerSimonian-Laird)

Pooled proportion = 0,006056 (95% CI = 0,002249 to 0,011697)

Bias indicators

Begg-Mazumdar: Kendall's tau = 0,911765 P < 0,0001

Egger: bias = 0,484164 (95% CI = 0,186125 to 0,782202) P = 0,0035

Harbord: bias = 2,49613 (92,5% CI = 0,823951 to 4,168309) P = 0,012

**Lagophtalmus**

Fascia lata

Non-combinability of studies

Cochran Q = 115,491061 (df = 18) P < 0,0001

Moment-based estimate of between studies variance = 0,08549

I² (inconsistency) = 84,4% (95% CI = 76,9% to 88,7%)

Random effects (DerSimonian-Laird)

Pooled proportion = 0,024602 (95% CI = 0,006729 to 0,053258)

Bias indicators

Begg-Mazumdar: Kendall's tau = 0,988304 P < 0,0001

Egger: bias = 1,1612 (95% CI = 0,055827 to 2,266572) P = 0,0406

Harbord: bias = 9,343051 (92,5% CI = 0,89084 to 17,795262) P = 0,0513

**Mersilene**

Non-combinability of studies

Cochran Q = 42,181125 (df = 11) P < 0,0001

Moment-based estimate of between studies variance = 0,084258

I² (inconsistency) = 73,9% (95% CI = 48,3% to 83,9%)

Random effects (DerSimonian-Laird)

Pooled proportion = 0,036008 (95% CI = 0,008489 to 0,081532)

Bias indicators

Begg-Mazumdar: Kendall's tau b = 0,753846 P = 0,0009

Egger: bias = 1,393733 (95% CI = 0,120238 to 2,667229) P = 0,0349

Harbord: bias = 1,519592 (92,5% CI = -4,270083 to 7,309268) P = 0,6134

PTFE

Non-combinability of studies

Cochran Q = 0,434134 (df = 4) P = 0,9796

Moment-based estimate of between studies variance = 0

I² (inconsistency) = 0% (95% CI = 0% to 64,1%)

Random effects (DerSimonian-Laird)

Pooled proportion = 0,003349 (95% CI = 0,000025 to 0,012231)

Bias indicators

Begg-Mazumdar: Kendall's error P > 0,9999 (low power)

Egger: bias = 0 (95% CI = 0 to 0) P = *

Harbord: bias = -1,49E-16 (92,5% CI = * to *) P < 0,0001

**Silicone**

Non-combinability of studies

Cochran Q = 80,733905 (df = 16) P < 0,0001

Moment-based estimate of between studies variance = 0,067707

I² (inconsistency) = 80,2% (95% CI = 68,1% to 86,3%)

Random effects (DerSimonian-Laird)

Pooled proportion = 0,022546 (95% CI = 0,006342 to 0,048361)

Bias indicators

Begg-Mazumdar: Kendall's tau = 0,852941 P < 0,0001

Egger: bias = 1,518329 (95% CI = 0,57681 to 2,459847) P = 0,0037

Harbord: bias = 3,436343 (92,5% CI = -3,261328 to 10,134015) P = 0,3419

**Asymmetry**

Fascia lata

Non-combinability of studies

Cochran Q = 69,772446 (df = 18) P < 0,0001

Moment-based estimate of between studies variance = 0,045399

I² (inconsistency) = 74,2% (95% CI = 57,2% to 82,5%)

Random effects (DerSimonian-Laird)

Pooled proportion = 0,020991 (95% CI = 0,007339 to 0,041447)

Bias indicators

Begg-Mazumdar: Kendall's tau = 0,567251 P = 0,0004

Egger: bias = 0,851028 (95% CI = -0,211625 to 1,913682) P = 0,1093

Harbord: bias = -1,911938 (92,5% CI = -3,945106 to 0,121231) P = 0,0924

Mersilene

Non-combinability of studies

Cochran Q = 22,925992 (df = 11) P = 0,0181

Moment-based estimate of between studies variance = 0,032227

I² (inconsistency) = 52% (95% CI = 0% to 73,6%)

Random effects (DerSimonian-Laird)

Pooled proportion = 0,023638 (95% CI = 0,006593 to 0,05084)

Bias indicators

Begg-Mazumdar: Kendall's tau b = 0,723077 P = 0,0015

Egger: bias = 0,960668 (95% CI = -0,09926 to 2,020595) P = 0,071

Harbord: bias = 2,61621 (92,5% CI = -2,422385 to 7,654804) P = 0,3265

PTFE

Non-combinability of studies

Cochran Q = 15,503097 (df = 4) P = 0,0038

Moment-based estimate of between studies variance = 0,045985

I² (inconsistency) = 74,2% (95% CI = 1,5% to 87,7%)

Random effects (DerSimonian-Laird)

Pooled proportion = 0,014671 (95% CI = 0,000097 to 0,05332)

Bias indicators

Begg-Mazumdar: Kendall's tau = 1 P = 0,0167 (low power)

Egger: bias = 1,123469 (95% CI = -1,2242 to 3,471139) P = 0,2251

Harbord: bias = 5,993093 (92,5% CI = -11,47407 to 23,460257) P = 0,4255

Silicone

Non-combinability of studies

Cochran Q = 57,983255 (df = 16) P < 0,0001

Moment-based estimate of between studies variance = 0,043911

I² (inconsistency) = 72,4% (95% CI = 51,6% to 81,9%)

Random effects (DerSimonian-Laird)

Pooled proportion = 0,016177 (95% CI = 0,004494 to 0,034921)

Bias indicators

Begg-Mazumdar: Kendall's tau = 0,764706 P < 0,0001

Egger: bias = 1,081106 (95% CI = 0,114718 to 2,047495) P = 0,0307

Harbord: bias = 0,027046 (92,5% CI = -5,830051 to 5,884144) P = 0,9931

**Granuloma**

Fascia lata

Non-combinability of studies

Cochran Q = 7,498643 (df = 18) P = 0,9852

Moment-based estimate of between studies variance = 0

I² (inconsistency) = 0% (95% CI = 0% to 42,9%)

Random effects (DerSimonian-Laird)

Pooled proportion = 0,004962 (95% CI = 0,001861 to 0,009544)

Bias indicators

Begg-Mazumdar: Kendall's tau = 0,684211 P < 0,0001

Egger: bias = 0,083427 (95% CI = -0,121866 to 0,28872) P = 0,4032

Harbord: bias = -0,203143 (92,5% CI = -0,868028 to 0,461742) P = 0,5699

Mersilene

Non-combinability of studies

Cochran Q = 17,453669 (df = 11) P = 0,0952

Moment-based estimate of between studies variance = 0,017439

I² (inconsistency) = 37% (95% CI = 0% to 66,9%)

Random effects (DerSimonian-Laird)

Pooled proportion = 0,022773 (95% CI = 0,007741 to 0,045463)

Bias indicators

Begg-Mazumdar: Kendall's tau b = 0,6875 P = 0,0029

Egger: bias = 0,451246 (95% CI = -1,101236 to 2,003729) P = 0,5318

Harbord: bias = -3,088013 (92,5% CI = -5,443232 to -0,732795) P = 0,0263

PTFE

Non-combinability of studies

Cochran Q = 8,857626 (df = 4) P = 0,0648

Moment-based estimate of between studies variance = 0,019419

I² (inconsistency) = 54,8% (95% CI = 0% to 81,3%)

Random effects (DerSimonian-Laird)

Pooled proportion = 0,012203 (95% CI = 0,00069 to 0,037591)

Bias indicators

Begg-Mazumdar: Kendall's tau = 0,6 P = 0,2333 (low power)

Egger: bias = 0,684557 (95% CI = -1,919058 to 3,288172) P = 0,4641

Harbord: bias = -1,739578 (92,5% CI = -9,924109 to 6,444952) P = 0,6087

Silicone

Non-combinability of studies

Cochran Q = 20,052798 (df = 16) P = 0,2179

Moment-based estimate of between studies variance = 0,004239

I² (inconsistency) = 20,2% (95% CI = 0% to 55,2%)

Random effects (DerSimonian-Laird)

Pooled proportion = 0,009974 (95% CI = 0,004226 to 0,018121)

Bias indicators

Begg-Mazumdar: Kendall's tau = 0,75 P < 0,0001

Egger: bias = 0,69313 (95% CI = 0,206037 to 1,180222) P = 0,0084

Harbord: bias = 1,668016 (92,5% CI = -0,811274 to 4,147306) P = 0,2175

**Herniation**

Fascia lata

Non-combinability of studies

Cochran Q = 10,009966 (df = 18) P = 0,9316

Moment-based estimate of between studies variance = 0

I² (inconsistency) = 0% (95% CI = 0% to 42,9%)

Random effects (DerSimonian-Laird)

Pooled proportion = 0,003572 (95% CI = 0,001053 to 0,007576)

Bias indicators

Begg-Mazumdar: Kendall's tau = 0,953216 P < 0,0001

Egger: bias = 0,107094 (95% CI = -0,082941 to 0,297129) P = 0,2508

Harbord: bias = 0,663538 (92,5% CI = -0,758066 to 2,085143) P = 0,3884

Mersilene

Non-combinability of studies

Cochran Q = 12,336727 (df = 11) P = 0,3389

Moment-based estimate of between studies variance = 0,003612

I² (inconsistency) = 10,8% (95% CI = 0% to 55,1%)

Random effects (DerSimonian-Laird)

Pooled proportion = 0,015356 (95% CI = 0,00524 to 0,030668)

Bias indicators

Begg-Mazumdar: Kendall's tau b = 0,784615 P = 0,0006

Egger: bias = 0,608638 (95% CI = 0,121483 to 1,095792) P = 0,0193

Harbord: bias = 1,882165 (92,5% CI = -0,56008 to 4,32441) P = 0,1567

PTFE

Non-combinability of studies

Cochran Q = 3,07376 (df = 4) P = 0,5456

Moment-based estimate of between studies variance = 0

I² (inconsistency) = 0% (95% CI = 0% to 64,1%)

Random effects (DerSimonian-Laird)

Pooled proportion = 0,004994 (95% CI = 0,000317 to 0,01521)

Bias indicators

Begg-Mazumdar: Kendall's tau = 1 P = 0,0167 (low power)

Egger: bias = 0,372801 (95% CI = -0,52792 to 1,273522) P = 0,2793

Harbord: bias = 1,556811 (92,5% CI = -2,980591 to 6,094213) P = 0,4255

Silicone

Non-combinability of studies

Cochran Q = 28,981399 (df = 16) P = 0,0241

Moment-based estimate of between studies variance = 0,013578

I² (inconsistency) = 44,8% (95% CI = 0% to 67,5%)

Random effects (DerSimonian-Laird)

Pooled proportion = 0,019977 (95% CI = 0,009782 to 0,033677)

Bias indicators

Begg-Mazumdar: Kendall's tau = 0,617647 P = 0,0003

Egger: bias = 1,247974 (95% CI = 0,748197 to 1,747751) P < 0,0001

Harbord: bias = 3,426185 (92,5% CI = 1,220461 to 5,631909) P = 0,0095

Abscessus

Fascia Lata

Non-combinability of studies

Cochran Q = 6,073086 (df = 18) P = 0,9959

Moment-based estimate of between studies variance = 0

I² (inconsistency) = 0% (95% CI = 0% to 42,9%)

Random effects (DerSimonian-Laird)

Pooled proportion = 0,004293 (95% CI = 0,00146 to 0,008609)

Bias indicators

Begg-Mazumdar: Kendall's tau = 0,836257 P < 0,0001

Egger: bias = 0,044235 (95% CI = -0,134584 to 0,223055) P = 0,6085

Harbord: bias = -0,25112 (92,5% CI = -0,790199 to 0,287959) P = 0,3893

Mersilene

Non-combinability of studies

Cochran Q = 3,187284 (df = 11) P = 0,988

Moment-based estimate of between studies variance = 0

I² (inconsistency) = 0% (95% CI = 0% to 49,8%)

Random effects (DerSimonian-Laird)

Pooled proportion = 0,00829 (95% CI = 0,001869 to 0,019227)

Bias indicators

Begg-Mazumdar: Kendall's tau b = 0,875 P = 0,0001

Egger: bias = 0,119505 (95% CI = -0,156663 to 0,395673) P = 0,3577

Harbord: bias = 0,128294 (92,5% CI = -0,974097 to 1,230684) P = 0,8218

PTFE

Non-combinability of studies

Cochran Q = 0,434134 (df = 4) P = 0,9796

Moment-based estimate of between studies variance = 0

I² (inconsistency) = 0% (95% CI = 0% to 64,1%)

Random effects (DerSimonian-Laird)

Pooled proportion = 0,003349 (95% CI = 0,000025 to 0,012231)

Bias indicators

Begg-Mazumdar: Kendall's error P > 0,9999 (low power)

Egger: bias = 0 (95% CI = 0 to 0) P = *

Harbord: bias = -1,49E-16 (92,5% CI = * to *) P < 0,0001

Silicone

Non-combinability of studies

Cochran Q = 8,467605 (df = 16) P = 0,9337

Moment-based estimate of between studies variance = 0

I² (inconsistency) = 0% (95% CI = 0% to 44,5%)

Random effects (DerSimonian-Laird)

Pooled proportion = 0,005455 (95% CI = 0,001888 to 0,010857)

Bias indicators

Begg-Mazumdar: Kendall's tau = 0,911765 P < 0,0001

Egger: bias = 0,283491 (95% CI = -0,049559 to 0,616541) P = 0,0897

Harbord: bias = 0,584878 (92,5% CI = -1,130681 to 2,300437) P = 0,5241

Amblyopia

Fascia Lata

Non-combinability of studies

Cochran Q = 365,960976 (df = 18) P < 0,0001

Moment-based estimate of between studies variance = 0,305128

I² (inconsistency) = 95,1% (95% CI = 93,9% to 95,9%)

Random effects (DerSimonian-Laird)

Pooled proportion = 0,020845 (95% CI = 0,000219 to 0,073719)

Bias indicators

Begg-Mazumdar: Kendall's tau = 0,883041 P < 0,0001

Egger: bias = 1,397249 (95% CI = -1,03483 to 3,829328) P = 0,242

Harbord: bias = -1,442677 (92,5% CI = -13,698129 to 10,812776) P = 0,826

Mersilene

Non-combinability of studies

Cochran Q = 1,105591 (df = 11) P > 0,9999

Moment-based estimate of between studies variance = 0

I² (inconsistency) = 0% (95% CI = 0% to 49,8%)

Random effects (DerSimonian-Laird)

Pooled proportion = 0,006605 (95% CI = 0,001117 to 0,016623)

Bias indicators

Begg-Mazumdar: Kendall's error P > 0,9999

Egger: bias = 0 (95% CI = 0 to 0) P = *

Harbord: bias = -6,95E-16 (92,5% CI = -2,29E-08 to 2,29E-08) P > 0,9999

PTFE

Non-combinability of studies

Cochran Q = 0,434134 (df = 4) P = 0,9796

Moment-based estimate of between studies variance = 0

I² (inconsistency) = 0% (95% CI = 0% to 64,1%)

Random effects (DerSimonian-Laird)

Pooled proportion = 0,003349 (95% CI = 0,000025 to 0,012231)

Bias indicators

Begg-Mazumdar: Kendall's error P > 0,9999 (low power)

Egger: bias = 0 (95% CI = 0 to 0) P = *

Harbord: bias = -1,49E-16 (92,5% CI = * to *) P < 0,0001

Silicone

Non-combinability of studies

Cochran Q = 1,356436 (df = 16) P > 0,9999

Moment-based estimate of between studies variance = 0

I² (inconsistency) = 0% (95% CI = 0% to 44,5%)

Random effects (DerSimonian-Laird)

Pooled proportion = 0,003808 (95% CI = 0,000979 to 0,008479)

Bias indicators

Begg-Mazumdar: Kendall's error P > 0,9999

Egger: bias = 0 (95% CI = 0 to 0) P = *

Harbord: bias = -9,81E-16 (92,5% CI = -7,70E-09 to 7,70E-09) P > 0,9999

Strabismus

Fascia Lata

Non-combinability of studies

Cochran Q = 58,609944 (df = 18) P < 0,0001

Moment-based estimate of between studies variance = 0,035611

I² (inconsistency) = 69,3% (95% CI = 46,7% to 79,7%)

Random effects (DerSimonian-Laird)

Pooled proportion = 0,012593 (95% CI = 0,003304 to 0,027761)

Bias indicators

Begg-Mazumdar: Kendall's tau = 0,894737 P < 0,0001

Egger: bias = 0,485339 (95% CI = -0,146052 to 1,11673) P = 0,1232

Harbord: bias = 0,668718 (92,5% CI = -3,422231 to 4,759666) P = 0,7603

Mersilene

Non-combinability of studies

Cochran Q = 1,105591 (df = 11) P > 0,9999

Moment-based estimate of between studies variance = 0

I² (inconsistency) = 0% (95% CI = 0% to 49,8%)

Random effects (DerSimonian-Laird)

Pooled proportion = 0,006605 (95% CI = 0,001117 to 0,016623)

Bias indicators

Begg-Mazumdar: Kendall's error P > 0,9999

Egger: bias = 0 (95% CI = 0 to 0) P = *

Harbord: bias = -6,95E-16 (92,5% CI = -2,29E-08 to 2,29E-08) P > 0,9999

PTFE

Non-combinability of studies

Cochran Q = 0,434134 (df = 4) P = 0,9796

Moment-based estimate of between studies variance = 0

I² (inconsistency) = 0% (95% CI = 0% to 64,1%)

Random effects (DerSimonian-Laird)

Pooled proportion = 0,003349 (95% CI = 0,000025 to 0,012231)

Bias indicators

Begg-Mazumdar: Kendall's error P > 0,9999 (low power)

Egger: bias = 0 (95% CI = 0 to 0) P = *

Harbord: bias = -1,49E-16 (92,5% CI = * to *) P < 0,0001

Silicone

Non-combinability of studies

Cochran Q = 1,356436 (df = 16) P > 0,9999

Moment-based estimate of between studies variance = 0

I² (inconsistency) = 0% (95% CI = 0% to 44,5%)

Random effects (DerSimonian-Laird)

Pooled proportion = 0,003808 (95% CI = 0,000979 to 0,008479)

Bias indicators

Begg-Mazumdar: Kendall's error P > 0,9999

Egger: bias = 0 (95% CI = 0 to 0) P = *

Harbord: bias = -9,81E-16 (92,5% CI = -7,70E-09 to 7,70E-09) P > 0,9999

Overcorrection

Fascia lata

Non-combinability of studies

Cochran Q = 53,195833 (df = 18) P < 0,0001

Moment-based estimate of between studies variance = 0,030863

I² (inconsistency) = 66,2% (95% CI = 39,8% to 78%)

Random effects (DerSimonian-Laird)

Pooled proportion = 0,012367 (95% CI = 0,003472 to 0,026628)

Bias indicators

Begg-Mazumdar: Kendall's tau = 0,777778 P < 0,0001

Egger: bias = 0,456145 (95% CI = -0,530407 to 1,442697) P = 0,343

Harbord: bias = -2,38819 (92,5% CI = -4,273888 to -0,502492) P = 0,028

Mersilene

Non-combinability of studies

Cochran Q = 8,705339 (df = 11) P = 0,6491

Moment-based estimate of between studies variance = 0

I² (inconsistency) = 0% (95% CI = 0% to 49,8%)

Random effects (DerSimonian-Laird)

Pooled proportion = 0,010082 (95% CI = 0,002769 to 0,021894)

Bias indicators

Begg-Mazumdar: Kendall's tau b = 0,9375 P < 0,0001

Egger: bias = 0,303895 (95% CI = -0,251946 to 0,859736) P = 0,2511

Harbord: bias = 0,349325 (92,5% CI = -2,652328 to 3,350978) P = 0,8218

PTFE

Non-combinability of studies

Cochran Q = 0,434134 (df = 4) P = 0,9796

Moment-based estimate of between studies variance = 0

I² (inconsistency) = 0% (95% CI = 0% to 64,1%)

Random effects (DerSimonian-Laird)

Pooled proportion = 0,003349 (95% CI = 0,000025 to 0,012231)

Bias indicators

Begg-Mazumdar: Kendall's error P > 0,9999 (low power)

Egger: bias = 0 (95% CI = 0 to 0) P = *

Harbord: bias = -1,49E-16 (92,5% CI = * to *) P < 0,0001

Silicone

Non-combinability of studies

Cochran Q = 10,726677 (df = 16) P = 0,826

Moment-based estimate of between studies variance = 0

I² (inconsistency) = 0% (95% CI = 0% to 44,5%)

Random effects (DerSimonian-Laird)

Pooled proportion = 0,007265 (95% CI = 0,003009 to 0,01335)

Bias indicators

Begg-Mazumdar: Kendall's tau = 0,720588 P < 0,0001

Egger: bias = 0,39277 (95% CI = 0,000852 to 0,784689) P = 0,0496

Harbord: bias = 0,796205 (92,5% CI = -0,904161 to 2,49657) P = 0,3845

Entropion

Fascia lata

Non-combinability of studies

Cochran Q = 47,470408 (df = 18) P = 0,0002

Moment-based estimate of between studies variance = 0,025843

I² (inconsistency) = 62,1% (95% CI = 30,5% to 75,8%)

Random effects (DerSimonian-Laird)

Pooled proportion = 0,019507 (95% CI = 0,008156 to 0,035599)

Bias indicators

Begg-Mazumdar: Kendall's tau = 0,590643 P = 0,0002

Egger: bias = 0,745579 (95% CI = 0,312656 to 1,178501) P = 0,0021

Harbord: bias = 2,425029 (92,5% CI = 0,271988 to 4,57807) P = 0,0475

Mersilene

Non-combinability of studies

Cochran Q = 1,105591 (df = 11) P > 0,9999

Moment-based estimate of between studies variance = 0

I² (inconsistency) = 0% (95% CI = 0% to 49,8%)

Random effects (DerSimonian-Laird)

Pooled proportion = 0,006605 (95% CI = 0,001117 to 0,016623)

Bias indicators

Begg-Mazumdar: Kendall's error P > 0,9999

Egger: bias = 0 (95% CI = 0 to 0) P = *

Harbord: bias = -6,95E-16 (92,5% CI = -2,29E-08 to 2,29E-08) P > 0,9999

PTFE

Non-combinability of studies

Cochran Q = 0,434134 (df = 4) P = 0,9796

Moment-based estimate of between studies variance = 0

I² (inconsistency) = 0% (95% CI = 0% to 64,1%)

Random effects (DerSimonian-Laird)

Pooled proportion = 0,003349 (95% CI = 0,000025 to 0,012231)

Bias indicators

Begg-Mazumdar: Kendall's error P > 0,9999 (low power)

Egger: bias = 0 (95% CI = 0 to 0) P = *

Harbord: bias = -1,49E-16 (92,5% CI = * to *) P < 0,0001

Silicone

Non-combinability of studies

Cochran Q = 4,847903 (df = 16) P = 0,9965

Moment-based estimate of between studies variance = 0

I² (inconsistency) = 0% (95% CI = 0% to 44,5%)

Random effects (DerSimonian-Laird)

Pooled proportion = 0,005132 (95% CI = 0,0017 to 0,010401)

Bias indicators

Begg-Mazumdar: Kendall's tau = 0,897059 P < 0,0001

Egger: bias = 0,107498 (95% CI = -0,204195 to 0,419191) P = 0,4736

Harbord: bias = -0,377247 (92,5% CI = -1,55054 to 0,796046) P = 0,5477

Hypertrophic scar

Fascia lata

Non-combinability of studies

Cochran Q = 24,372912 (df = 18) P = 0,1432

Moment-based estimate of between studies variance = 0,005588

I² (inconsistency) = 26,1% (95% CI = 0% to 57%)

Random effects (DerSimonian-Laird)

Pooled proportion = 0,008339 (95% CI = 0,0032 to 0,015866)

Bias indicators

Begg-Mazumdar: Kendall's tau = 0,74269 P < 0,0001

Egger: bias = 0,382299 (95% CI = -0,048122 to 0,81272) P = 0,0782

Harbord: bias = 2,003222 (92,5% CI = -0,247562 to 4,254007) P = 0,1097
