# Supplementary material for: Suspensory Materials for Surgery of Blepharoptosis: A Systematic Review of Observational Studies
Source: PLoS One. 2016 Sep 15;11(9):e0160827. doi: 10.1371/journal.pone.0160827 (PMC5025102; doi:10.1371/journal.pone.0160827)
Supplement: S1 Table — (DOCX) [file pone.0160827.s003.docx]

| First author (Year) | Title | Motivation |
| --- | --- | --- |
| Ahn (2008) | Frontalis sling operation using silicone rod for the correction of ptosis in chronic progressive external ophthalmoplegia | Study design |
| Alagoz (2008) | Morphologic assessment of the tendon graft donor sites: palmaris longus, plantaris, tensor fascia lata | Off topic |
| Allen (2015) | Factors affecting eyelid crease formation before and after silicone frontalis suspension for adult-onset myogenic ptosis | Off topic |
| Arnaud (1989) | [Use of Gore-Tex in ptosis surgery for suspension from the frontal muscle] | Not available in english |
| Arslan (2004) | Enhanced frontalis sling with double-fixed, solvent-dehydrated cadaveric fascia lata allograft in the management of eye ptosis | Off topic |
| Badakere (2013) | Re: "The 18-gauge needle: an innovative simple tool for frontalis sling surgery" | Study design |
| Bahmani-Kashkouli (2008) | A novel technique of small incision fascia lata harvesting without a faciatome for frontalis suspension procedure | Off topic |
| Bajaj (2004) | Evaluation of polytetrafluoroethylene suture for frontalis suspension as compared to polybutylate-coated braided polyester | Materials used in surgical operations |
| Baker (2005) | A novel technique of harvesting temporalis fascia autografts for correction of recurrent blepharoptosis | Study design |
| Barberini (1992) | [Congenital ptosis or blepharoptosis of the superior palpebrae: surgical correction by transplant of the fascia lata] | Not available in english |
| Baroody (2005) | Advances in the diagnosis and treatment of ptosis | Study design |
| Bartley (2002) | The enhanced frontalis sling for blepharoptosis repair | Study design |
| Ben Simon (2005) | Frontalis suspension for upper eyelid ptosis: evaluation of different surgical designs and suture material | Materials used in surgical operations |
| Bernardini (2007) | Treatment of Unilateral Congenital Ptosis | Study design |
| Bernardini (2010) | Re: "Outcomes in silicone rod frontalis suspension surgery for high-risk noncongenital blepharoptosis" | Study design |
| Bernardini (2013) | Treatment of unilateral congenital ptosis: putting the debate to rest | Study design |
| Berry-Brincat (2009) | Paediatric blepharoptosis: a 10-year review | Materials used in surgical operations |
| Bladen (2012) | The use of a pleated strip of autogenous temporalis fascia graft for frontalis suspension in recurrent poor levator function ptosis in adult patients | Materials used in surgical operations |
| Bleyen (2009) | Muscle prolapse after harvesting autogenous fascia lata used for frontalis suspension in children | Complications not described |
| Bouazza (2014) | [Treatment of congenital ptosis by frontalis suspension with monofilament polypropylene suture: results of a study of 21 cases] | Not available in english |
| Bruun (1991) | [Ptosis operations with silicone suspension at the eyebrow] | Not available in english |
| Cadera (1992) | Changes in astigmatism after surgery for congenital ptosis | Off topic |
| Caputo (1989) | An alternative needle for frontalis suspension | Off topic |
| Cartwright (1995) | Microdeletion of chromosome 7P syndrome ocular manifestations | Off topic |
| Chait (1989) | A modified frontalis sling in the treatment of combined blepharoptosis and facial nerve paresis | Study design |
| Choi (2011) | Nanostructural investigation of frontalis sling biomaterial surfaces | Materials used in surgical operations |
| Dailey (1991) | Transconjunctival frontalis suspension (TCFS) | Off topic |
| D'Aniello (2000) | Autologous fascial graft in the treament of third degree congenital blepharoptosis [L'innesto autologo di fascia lata nel trattamento della blefaroptosi congenita di terzo grado] | Not available in english |
| Debski (2012) | Frontalis suspension using autogenous fascia lata - Evaluation of long-term outcome [Podwieszenie powiek górnych na paskach powiezi szerokiej uda - ocena późnych wyników] | Not available in english |
| D'Esposito (1989) | Ptosis correction in the context of the treatment of external congenital ophthalmoplegia | Off topic |
| Detorakis (2009) | Successful conservative management of expanded polytetrafluoroethylene band exposure in frontalis suspension | Study design |
| Donker (2005) | Blepharoptosis and upper eyelid swelling due to lipogranulomatous inflammation caused by silicone oil | Study design |
| Downes (1989) | The Mersilene mesh sling--a new concept in ptosis surgery | Study design |
| Egbert (2001) | Alternating ptosis after bilateral frontalis muscle suspension for congenital ptosis | Study design |
| Ellis (1993) | Assessment and treatment of the paralyzed lower eyelid | Off topic |
| Emekli (1996) | Chronic progressive external ophthalmoplegia [KRONIK PROGRESIF EKSTERNAL OFTALMOPLEJI] | Not available in english |
| Esmaeli (1998) | Long-term results of frontalis suspension using irradiated, banked fascia lata | Materials used in surgical operations |
| Fry (2011) | Re: "Muscle prolapse after harvesting autogenous fascia lata used for frontalis suspension in children" | Study design |
| Fry (2012) | Re: "Re: Muscle prolapse after harvesting autogenous fascia lata for frontalis suspension in children," by Fry and Naugle | Study design |
| Fujita (2013) | A ringed fascia lata graft without peritendinous areolar tissue encircling the levator veli palatini and superior pharyngeal constrictor muscles gradually shrinks to reduce velopharyngeal incompetence, functioning as an intravelar palatal lift | Off topic |
| Gawdat (2010) | Evaluation of transconjunctival frontalis suspension using nonabsorbable suture | Off topic |
| Gazzola (2011) | Congenital ptosis and blefarophimosis: retrospective analysis of the effectiveness of correction with levator resection and frontalis suspension | Materials used in surgical operations |
| Goel (2012) | The 18-gauge needle: an innovative simple tool for frontalis sling surgery | Complications not described |
| Goel (2013) | Reply Re: "The 18-gauge needle: an innovative simple tool for frontalis sling surgery" | Study design |
| Goldberger (1991) | Double rhomboid silicone rod frontalis suspension | Study design |
| Green (1997) | Removal of an infected silicone rod frontalis sling without recurrence of ptosis | Study design |
| Gresly (1992) | [Unilateral eyebrow suspension in severe unilateral congenital ptosis] | Not available in english |
| Gundeslioglu (2012) | Correction of severe ptosis with a silicone implant suspensor: 22 years of experience | Study design |
| Gundeslioglu (2013) | An frontalis sling operation using an autogenous en-bloc, fan-shaped tensor fascia lata graft for blepharoptosis | Study design |
| Guntinas-Lichius (2009) | [Reconstructive surgery for patients with facial palsy] | Not available in english |
| Gürdal (2003) | Autogenous versus allograft fascia lata in frontal sling surgery --long-term results | Complications not described |
| Hague (1994) | Blepharoplasty and ptosis | Study design |
| Hersh, Dov (2006) | Comparison of silastic and banked fascia lata in pediatric frontalis suspension | Materials used in surgical operations |
| Hostovsky (2010) | Orbital inflammation as a late complication of frontalis silicone elastomer sling operation for congenital ptosis | Study design |
| Ibrahim (2015) | The use of Whitnall's ligament for sling redirection in frontalis suspension ptosis surgery | Materials used in surgical operations |
| Ichinose (2010) | Congenital blepharoptosis co-occurring with VATER association | Off topic |
| Imai (2013) | Frontalis suspension with single rhomboid-shaped fascial strip in severe congenital blepharoptosis; Our procedure and eyelid positioning | full text not available |
| Isik (2013) | Effect of brow lifting using botulinum a toxin on upper eyelid height in patients with ptosis undergoing the frontal sling technique | Off topic |
| Jeong (2000) | Histopathological study of frontalis suspension materials | Off topic |
| Junceda-Moreno (2005) | [Treatment of palpebral ptosis with frontal suspension: a comparative study of different materials] | Not available in english |
| Kashkouli (2007) | A novel technique for small-incision fascia lata harvesting without a fasciatome for the frontalis suspension procedure | Complications not described |
| Kashkouli (2008) | Re: "a randomized clinical trial of two methods of fascia lata suspension in congenital ptosis" | Study design |
| Kashkouli (2014) | Re: "Evaluation of safety and efficacy of silicone rod in tarsofrontalis sling surgery for severe congenital ptosis" | Study design |
| Kataev (2006) | [Childhood ophthalmoplasty] | Not available in english |
| Kim (2012) | Prediction of postoperative eyelid height after frontalis suspension using autogenous fascia lata for pediatric congenital ptosis | Complications not described |
| Kobus (2008) | Analysis of treatment results 389 patients with congenital blepharoptosis [Analiza wyników leczenia 389 chorych z wrodzonym opadaniem powiek] | Not available in english |
| Kobus (2008) | Orbitoblepharophimosis syndrome - Own clinical experience in treatment of 60 patients [Zespół małoocza - Własne doświadczenia w leczeniu 60 pacjentów] | Not available in english |
| Kuchar (1997) | [Frontalis suspension with "expanded polytetrafluoroethylene (ePTFE) strips" in congenital ptosis] | Not available in english |
| Kumar (2005) | Clinical evaluation of refractive changes following brow suspension surgery in pediatric patients with congenital blepharoptosis | Off topic |
| Kwon (2014) | Microstructure and mechanical properties of synthetic brow-suspension materials | Study design |
| Lam (1996) | Palmaris longus tendon as a new autogenous material for frontalis suspension surgery in adults | Materials used in surgical operations |
| Lam (1998) | Autogenous palmaris longus tendon as frontalis suspension material for ptosis correction in children | Off topic |
| Lee (2002) | Aetiology and surgical treatment of childhood blepharoptosis | Off topic |
| Lee (2005) | Polytetrafluoroethylene as a spacer graft for the correction of lower eyelid retraction | Off topic |
| Lim (1998) | The use of sodium hyaluronate as a lubricant in brow suspension ptosis surgery using polyester fiber mesh | Off topic |
| Liu (1994) | Surgical correction of ptosis in ocular fibrosis syndrome | Study design |
| Liu (2011) | Comparative study on the three surgical procedures for severe congenital ptosis | full text not available |
| Louis (2013) | [Ptosis secondary to cavernous sinus meningioma] | Not available in english |
| Louis (2013) | [Ptosis secondary to cavernous sinus meningioma]. [Ptosis révélant un méningiome du sinus caverneux.] | Not available in english |
| Malhotra (2007) | Endoscopic harvesting of autogenous fascia lata | Off topic |
| Malhotra (2011) | Re: "Muscle prolapse after harvesting autogenous fascia lata used for frontalis suspension in children" | Study design |
| Manners (1994) | The use of Prolene as a temporary suspensory material for brow suspension in young children | Materials used in surgical operations |
| Marcoli (1997) | [Simplification in locating and dissecting the levator muscle of the upper eyelid in surgery for ptosis] | Not available in english |
| Marsh (2000) | Ptosis repair in a patient with oculopharyngeal dystrophy: brow suspension using autogenous fascia lata by spinal anaesthesia | Study design |
| Matsuo (2009) | Frontalis suspension with fascia lata for severe congenital blepharoptosis using enhanced involuntary reflex contraction of the frontalis muscle | Off topic |
| Mauriello (1998) | Effectiveness of homologous cadaveric fascia lata and role of suture fixation to tarsus in frontalis suspension | Off topic |
| Mehta (2004) | Functional results and complications of Mersilene mesh use for frontalis suspension ptosis surgery | Study design |
| Mencía-Gutiérrez (2005) | [Results and complications of expanded polytetrafluoroethylene in frontalis suspension ptosis surgery. Study of 59 cases] | Not available in english |
| Merbs (2003) | A Review of Common Eyelid Conditions for the Primary Care Physician | Study design |
| Molina-Terrazas (1997) | Surgical treatment of severe ptosis. Fascia lata frontal fixation: A 20 cases report [Manejo quirurgico de ptosis severa. Fijacion al frontal con fascia lata. Reporte de 20 casos] | Not available in english |
| Morax (1989) | Choice of treatment in surgery for congenital ptosis [CHOIX D'UN TRAITEMENT DANS LA CHIRURGIE DU PTOSIS CONGENITAL] | Not available in english |
| Morax (1991) | Temporalis fascia suspension in the treatment of ptosis | Materials used in surgical operations |
| Morax (1992) | Orbito-palpebral reconstruction in anophthalmos and severe congenital microphthalmos | Off topic |
| Morris (2008) | Safety and efficacy of silicone rod frontalis suspension surgery for childhood ptosis repair | Complications not described |
| Moscato (2011) | Frontalis suspension for the correction of blepharoptosis | Study design |
| Mutlu (1999) | Extrusion and granuloma formation with mersilene mesh brow suspension | Study design |
| Naugle (1997) | High leg incision fascia lata harvesting | Complications not described |
| Naugle (1998) | Complications of fascia lata harvesting for ptosis surgery | Study design |
| Naugle (1999) | Autogenous palmaris longus tendon as frontalis suspension material for ptosis correction in children (multiple letters) | Off topic |
| No authors listed (1994) | [Oculoplastic surgery: the choice of materials in palpebral suspension and eyelid lengthening. Comparison between autologous flaps and biomaterials] | Not available in english |
| No authors listed (2009) | My personal approach to surgery andprevention of surgical complications ; Frontalis suspension with Fascia lata for cogenital blepharoptosis | Study design |
| Noguchi (2011) | Tendon grafts with peritendinous areolar tissue increase the survival of endotendinous tissues and diminish postoperative shrinkage | Off topic |
| O'Reilly (1998) | Congenital ptosis: longterm results using stored fascia lata | Materials used in surgical operations |
| Osborne (2011) | Modified eyelid crease approach frontalis suspension without brow incision | Study design |
| Park (2008) | Results of long-term follow-up observations of blepharoptosis correction using the palmaris longus tendon | Materials used in surgical operations |
| Partyngl (1991) | [Lower eyelid extension-plasty in patients with ptosis in chronic progressive external ophthalmoplegia] | Not available in english |
| Pfeiffer (2012) | [Optimised frontal suspension for correction of complicated ptosis] | Not available in english |
| Putterman (1989) | Custom orbital implant in the repair of late posttraumatic enophthalmos | Off topic |
| Quintyn (2003) | Silicone oil migration in the eyelid after vitrectomy for retinal detachment | Off topic |
| Rama (1997) | Silicone frontalis slings for correction of blepharoptosis | Study design |
| Rizvi (2014) | Reply re: "Evaluation of safety and efficacy of silicone rod in tarsofrontalis sling surgery for severe congenital ptosis" | Study design |
| Ruban (1995) | [A new biomaterial in surgery of ptosis with frontalis suspension: wide pore PTFE] | Not available in english |
| Santaella (2011) | Carbon dioxide laser-induced combustion of extravasated intraocular silicone oil in the eyelid mimicking xanthelasma | Off topic |
| Sebastiá (2011) | A one-stage correction of the blepharophimosis syndrome using a standard combination of surgical techniques | Off topic |
| Sebastiá (2015) | Bilateral lid/brow elevation procedure for severe ptosis in Kearns-Sayre syndrome, a mitochondrial cytopathy | Study design |
| Seider (2006) | One medial triangular Tutoplast sling as a frontalis suspension for adult myogenic blepharoptosis | Materials used in surgical operations |
| Şekeroğlu (2015) | Silicone rod exposure after frontalis suspension procedure for blepharoptosis [Askılama yöntemi ile pitozis cerrahisi yapılan olgularda silikon materyal atılımı] | Study design |
| Shinder (2012) | Correction of severe ptosis with a silicone implant suspensor | Study design |
| Silvério (2009) | [Frontalis suspension with polytetrafluorethylene for the treatment of blepharoptosis] | Not available in english |
| Spoor (1990) | Blepharoptosis repair by fascia lata suspension with direct tarsal and frontalis fixation | Study design |
| Sredzińska-Kita (2013) | [Surgical management of congenital eyelid ptosis with silicone rod--outcome analysis] | Not available in english |
| Sredzińska-Kita (2014) | [Visual function assessment in children after surgical management of congenital eyelid ptosis using the silicone rod] | Not available in english |
| Takahashi (2010) | Frontalis suspension surgery in upper eyelid blepharoptosis | Study design |
| Tamir (1990) | [Oculopharyngeal muscular dystrophy] | Not available in english |
| Tanenbaum (2014) | Frontalis suspension with supramid suture: longevity results in very young patients with congenital ptosis | Materials used in surgical operations |
| Taylor (2007) | Blepharophimosis-ptosis-epicanthus inversus syndrome: objective analysis of surgical outcome in patients from a single unit | Off topic |
| Teske (1996) | Congenital ptosis: results of surgical management | full text not available |
| Tsai (2002) | Use of the orbicularis oculi muscle flap for severe Marcus Gunn ptosis | Off topic |
| Wagner (2006) | The search for an ideal surgical procedure for pediatric congenital ptosis with poor to absent levator muscle function continues | Study design |
| Wagner (2015) | Comparing suture materials used in frontalis suspension procedures | Study design |
| Wang (1994) | Use of Gore-Tex sling in brow suspension procedure | full text not available |
| Wheatcroft (1997) | Complications of fascia lata harvesting for ptosis surgery | Complications not described |
| Whitehouse (1995) | Congenital ptosis: results of surgical management | full text not available |
| Wilson (1991) | Congenital ptosis. Long-term results of treatment using lyophilized fascia lata for frontalis suspensions | Materials used in surgical operations |
| Wobig (1989) | Surgical technique for ptosis repair | Study design |
| Wojno (2000) | Sling removal | Study design |
| Wong (2002) | Management of myogenic ptosis | Off topic |
| Wong (2005) | Long-term results of autogenous palmaris longus frontalis sling in children with congenital ptosis | Materials used in surgical operations |
| Yagci (2003) | Comparison of cosmetic results in frontalis sling operations: the eyelid crease incision versus the supralash stab incision | Off topic |
| Yoon (2008) | Bell's phenomenon protects the tear film and ocular surface after frontalis suspension surgery for congenital ptosis | Off topic |
| Zweep (1992) | Evaluation of expanded polytetrafluoroethylene (e-PTFE) and autogenous fascia lata in frontalis suspension. A comparative clinical study | full text not available |
